# From FAIR to CURE: guidelines for computational models of biological systems

**DOI:** 10.1038/s41540-026-00651-0

**Published:** 2026-03-27

**Authors:** Herbert M. Sauro, Eran Agmon, Michael L. Blinov, John H. Gennari, Joseph L. Hellerstein, Adel Heydarabadipour, Bartholomew E. Jardine, Elebeoba May, David P. Nickerson, Lucian P. Smith, Gary D. Bader, Frank T. Bergmann, Patrick M. Boyle, Andreas Dräger, James R. Faeder, Song Feng, Juliana Freire, Fabian Fröhlich, James A. Glazier, Thomas E. Gorochowski, Tomas Helikar, Henning Hermjakob, Stefan Hoops, Peter Hunter, Princess I. Imoukhuede, Sarah M. Keating, Matthias König, Reinhard Laubenbacher, Leslie M. Loew, Carlos F. Lopez, William W. Lytton, Rahuman S. Malik-Sheriff, Andrew McCulloch, Pedro Mendes, Lealem Mulugeta, Chris J. Myers, Jerry G. Myers Jr, Anna Niarakis, David D. van Niekerk, Brett G. Olivier, Alexander A. Patrie, Ellen M. Quardokus, Nicole Radde, Johann M. Rohwer, Sven Sahle, James C. Schaff, Falk Schreiber, T. J. Sego, Janis Shin, Jacky L. Snoep, Rajanikanth Vadigepalli, H. Steven Wiley, Dagmar Waltemath, Ion I. Moraru

**Affiliations:** 1 Department of Bioengineering, University of Washington, Seattle, WA USA; 2 eScience Institute, University of Washington, Seattle, WA USA; 3 Center for Cell Analysis and Modeling, UConn Health, Farmington, CT USA; 4 Department of Biomedical Informatics & Medical Education, University of Washington, Seattle, WA USA; 5 Wisconsin Institute for Discovery, University of Wisconsin–Madison, Madison, WI USA; 6 Auckland Bioengineering Institute, University of Auckland, Auckland, New Zealand; 7 The Donnelly Centre, University of Toronto, Toronto, ON Canada; 8 BioQUANT, Heidelberg University, Heidelberg, Germany; 9 Center for Cardiovascular Biology, University of Washington, Seattle, WA USA; 10 Institute for Stem Cell and Regenerative Medicine, University of Washington, Seattle, WA USA; 11 Martin Luther University Halle-Wittenberg, Data Analytics and Bioinformatics, Halle, Germany; 12 Department of Computational and Systems Biology, University of Pittsburgh, Pittsburgh, PA USA; 13 Biological Sciences Division, Pacific Northwest National Laboratory, Richland, WA USA; 14 Department of Computer Science and Center for Data Science, New York University, New York, NY USA; 15 Dynamics of Living Systems Laboratory, The Francis Crick Institute, London, UK; 16 Intelligent Systems Engineering and Biocomplexity Institute, Indiana University, Bloomington, IN USA; 17 School of Biological Sciences, University of Bristol, Bristol, UK; 18 Department of Biochemistry, University of Nebraska-Lincoln, Lincoln, NE USA; 19 European Molecular Biology Laboratory, European Bioinformatics Institute (EMBL-EBI), Hinxton, Cambridgeshire UK; 20 Biocomplexity Institute, University of Virginia, Charlottesville, VA USA; 21 Advanced Research Computing Centre, University College London, London, UK; 22 Faculty of Life Science, Institute for Biology, Systems Medicine of Liver, Humboldt-University Berlin, Berlin, Germany; 23 Department of Medicine, University of Florida, Gainesville, FL USA; 24 Multiscale Modeling Group, Altos Labs, Redwood City, CA USA; 25 Departments of Physiology & Pharmacology, Neurology, Downstate Health Science University, Brooklyn, NY USA; 26 Department of Neurology, Kings County Hospital, Brooklyn, NY USA; 27 Departments of Bioengineering and Medicine, University of California San Diego, La Jolla, CA USA; 28 InSilico Labs LLC, Houston, TX USA; 29 Medalist Performance, Houston, TX USA; 30 Department of Electrical, Computer, and Energy Engineering, University of Colorado Boulder, Boulder, CO USA; 31 NASA - Glenn Research Center, Cleveland, OH USA; 32 Molecular, Cellular and Developmental Biology Unit (MCD), Center of Integrative Biology, University of Toulouse III-Paul Sabatier, Toulouse, France; 33 Lifeware Group, Inria, Palaiseau, France; 34 Department of Biochemistry, University of Stellenbosch, Matieland, South Africa; 35 Amsterdam Institute for Life and Environment, Vrije Universiteit Amsterdam, Amsterdam, Netherlands; 36 Institute for Stochastics and Applications, University of Stuttgart, Germany, Germany; 37 Department of Computer and Information Science, University of Konstanz, Konstanz, Germany; 38 Faculty of Information Technology, Monash University, Melbourne, VIC Australia; 39 Department of Pathology and Genomic Medicine, Thomas Jefferson University, Philadelphia, PA USA; 40 Environmental Molecular Sciences Laboratory, Pacific Northwest National Laboratory, Richland, WA USA; 41 Medical Informatics Laboratory, University Medicine Greifswald, Greifswald, Germany

**Keywords:** Computational biology and bioinformatics, Systems biology, Standardization

## Abstract

Guidelines for managing scientific data have been established under the FAIR principles, requiring that data be Findable, Accessible, Interoperable, and Reusable. In many scientific disciplines, especially computational biology, both data and models are key to progress. For this reason, and recognizing that such models are a very special type of “data”, we argue that computational models, especially mechanistic models prevalent in medicine, physiology and systems biology, deserve a complementary set of guidelines. We propose the CURE principles, emphasizing that models should be Credible, Understandable, Reproducible, and Extensible. We delve into each principle, discussing verification, validation, and uncertainty quantification for model credibility; the clarity of model descriptions and annotations for understandability; adherence to standards and open science practices for reproducibility; and the use of open standards and modular code for extensibility and reuse. We outline recommended and baseline requirements for each aspect of CURE, aiming to enhance the impact and trustworthiness of computational models, particularly in biomedical applications where credibility is paramount. Our perspective underscores the need for a more disciplined approach to modeling, aligning with emerging trends such as Digital Twins and emphasizing the importance of data and modeling standards for interoperability and reuse. Finally, we emphasize that given the non-trivial effort required to implement the guidelines, the community should strive to automate as many of the guidelines as possible.

## Introduction

Wilkinson et al.^1^ made in 2016 a good case (dare we say a fair case) for establishing guidelines for the management of scientific data. They arrived at four guiding principles enshrined in the acronym FAIR, namely that data be Findable, Accessible, Interoperable, and Reusable. With the rapid growth of computational modeling, especially the development of mechanistic models of physiological and cellular systems, the question arises of how these principles can be extended so that they can succinctly describe best practices for model management (e.g., model development, deployment, and interpretation). In this perspective, we introduce a set of complementary guidelines to FAIR that address the specific needs for mechanistic models. We identify four principles: Credibility, Understandability, Reproducibility, and Extensibility. We refer to these as the **CURE** principles.

We focus on mechanistic models, thereby excluding the growing body of machine-learning (ML) and AI models that are based solely on data. The machine learning community has appropriately encouraged the use of FAIR principles when publishing ML models^2,3^, with an emphasis on ensuring that data are accessible to those who wish to repeat the study and that models are explainable. However, the reproducibility of these models is a separate topic that has its own special concerns. Thus, we will not consider ML/AI models in this perspective.

Although the focus of this proposal is on models from the systems biology and systems medicine community, the guidelines and sentiments we describe are broadly applicable to other modeling domains.

## Why have guidelines?

Models are indispensable in many scientific and engineering disciplines. Examples include circuit simulation in electrical engineering, models of fluid flow in mechanical engineering, and weather modeling in atmospheric science. In some cases, modeling has progressed to the development of Digital Twins^4,5^, in which a simulation model is designed to replicate and interact with the physical system, or virtual populations, which can be used for applications such as clinical trial design^6^. Biological modeling has not yet risen to that same level of sophistication, with the possible exception of areas such as protein folding^7^ and molecular dynamics^8^ where physics and chemistry play a more important role. Even so, biological modeling is a rapidly evolving field. As the field grows, guidelines in the spirit of FAIR will help modelers create more impactful and credible models. We believe that these guidelines will be of importance for models that ultimately reach the clinic, and particularly for the growing interest in developing biomedical Digital Twins^9,10^ or whole-cell models^11–13^. The Working Group ‘Building Immune Digital Twins’ tries to address these questions, with a focus on the human immune system and its responses in various pathological contexts^9,10^.

## Who will use the guidelines?

Not all groups may need to use the CURE guidelines. For example, researchers who create conceptual models as part of a basic research project are unlikely to use guidelines. For academic researchers who build models of real systems, the choice has to be made by the researcher. At minimum, we recommend that a published model should at least be reproducible^14–17^. This does not necessarily mean the use of open standards, but models that do not use open standards should at least be well documented and annotated. The use of open standards would allow the model to be stored on one of the open model repositories, as well as making the model much more reusable, but ultimately, this decision is at the discretion of the researcher. For models that may eventually reach the clinic, the use of the CURE guidelines is highly recommended. For those involved in building large models, such as Digital Twins^9,10^ or Whole-cell models^11–13^, we highly recommend considering the use of the CURE guidelines.

## Existing guidelines

Several groups have proposed guidelines to improve best practices in creating biological models over the last 20 years. Of particular note is the creation of standardized languages for biological models^18^ such as Systems Biology Markup Language (SBML)^19,20^, CellML^21^, and NeuroML^22^. These are machine-readable formats that are an explicit representation of the model. By explicit representation, we mean that the model representation only includes elements that are essential for the model; it does not include implementation details related to simulation. For example, the essential characteristics of a mechanistic model of a well-mixed biochemical system include chemical species, reactions, and rate laws. It does not include software details such as file input/output and control logic for numerical solvers. An explicit representation is independent of its implementation in software.

The choice of an explicit representation for models is driven by the requirements of the communities that develop and use the models. SBML focuses on biochemical models where the representation is in terms of the biological processes. CellML focuses on a mathematical representation of models as differential-algebraic equations. NeuroML focuses on representations of neural systems. These representations have become popular among modelers and software developers. For example, all genome-scale models^23^ are represented using SBML, and thousands of kinetic models are now stored on publicly accessible model repositories using these formats^24–26^. Standards such as SBML avoid the use of potentially confusing and unreusable ad hoc models, allowing models to persist in a reproducible form over long periods of time^27,28^. Many authors, however, still publish models in executable formats such as MATLAB, Python, etc., which can pose problems for reproducibility and reuse, particularly when poorly documented^15,29^. The logical modeling community, which uses SBML Qualitative format (SBML-Qual) to encode logical and Boolean models^30^, made efforts to define a roadmap toward the annotation and curation of logical models (also known as the CALM initiative), including milestones for best practices and recommended standard requirements^31^.

To promote data sharing and reuse, the FAIR principles recommend a data dictionary that specifies data types and semantics for data items^1^. An analogous requirement exists for models. For example, consider an SBML model with the reaction *A* → *B*. Annotations are used to define *A*, *B*, and provide information about the reaction (e.g., the organism, cell type, and organelle in which the reaction takes place). Annotation can provide additional ontological and reference information about a model, including its submodels, processes, assumptions, and provenance. Genome-scale models are heavily annotated with process metadata^32,33^, and the curators at BioModels^24^ regularly add annotations to curated models. Guidelines for harmonizing semantic annotations for computational models have been established^34^. As part of these efforts, the systems biology and physiology community developed the MIRIAM standard, which describes the “Minimum Information Required In the Annotation of Models”^35^ and the OMEX metadata specification^34^. MIRIAM applies to structured information, such as SBML or CellML, where annotation information can be included in a machine-readable manner. Such information can be of great utility for searching, merging, or disassembling a model into its component parts^36^.

## Mechanistic models

The focus of this perspective is on mechanistic models. Such models are sometimes called deductive models in contrast to inductive models, which are purely data-driven^37^. We define a mechanistic model as: a representation of a biological system that is described in terms of the constituent physical parts and processes that occur between the parts. For example, in a mechanistic model of a cell signaling pathway, we would specify the various proteins and their phosphorylation states and the transformations between these states via enzymatic processes involving kinases and phosphatases.

Often, a mechanistic model is transformed into a computational model by invoking physical laws, such as the conservation of mass and chemical kinetic laws that govern individual transformations. In physiology and systems biology, models are commonly represented as a system of ordinary differential equations^38,39^, but other representations are also used, such as systems of Boolean functions, graphs, stochastic systems, and constraint-based models^40^. Such models are often shared via model repositories such as BioModels^24^, JWS Online^41^, or BiGG^25^ and KBase^26^ for constraint-based models.

Models can also be shared using raw executable formats such as Python or MATLAB that describe differential equations. Such ‘raw’ model code is typically provided as supplementary files to a published paper or stored in code repositories such as GitHub or specialized repositories such as ModelDB^42^. In some cases, the model may be described as part of the main text of the published article^43^ or be absent altogether. This obviously makes the reproducibility of such work much more complex and sometimes impossible, given the frequency of typographical errors in printing mathematical equations or code fragments. Models-as-programs paradigms, such as those followed by PySB^44^ and others, encourage the use of best practices in Python coding and documentation through tools such as Sphinx^45^, but these approaches rely on developer effort to document the code and model at an appropriate level of detail. They also tie a model to a specific language implementation, making reuse difficult and backward compatibility a problem, though PySB does offer some SBML support.

It may be difficult to abstract the underlying physiological processes of some complex systems purely in explicit form, though there have been few efforts to attempt this. Multi-scale modeling tends to give rise to such challenging scenarios. The context of computational modeling of electrophysiological phenomena in the heart provides an illustrative example^46^. Ordinary differential equations describing cell-scale events like ion channel gating and the generation of action potentials can be encoded using CellML or as a biological description in SBML. However, representing the propagation of excitatory wavefronts requires spatial discretization of the governing partial differential equations via finite element analysis; explicit formats for such applications exist^47,48^ but have not seen widespread use. One approach to improving interoperability and reproducibility is to create tools for importing model components written in common data formats. For instance, the openCARP simulation environment^46^ includes a CellML “translator” that facilitates on-the-fly incorporation of cell-scale representations of different types of cardiac electrophyiology (e.g., cardiac region, species, or disease-specific action potentials) within the fabric of the multi-scale simulation ecosystem.

## The CURE guidelines

**Fig. 1:**
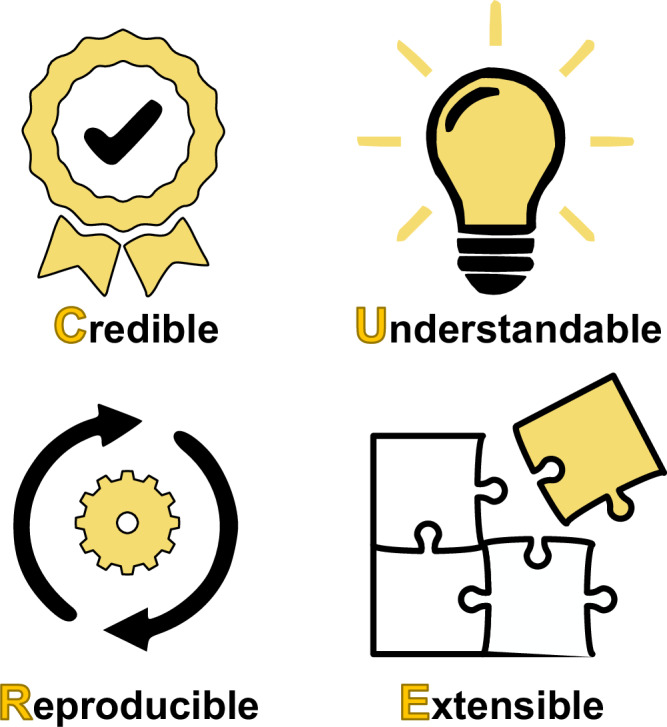
Schematic representation of the CURE guidelines. The guidelines emphasize Credibility, Understandability, Reproducibility, and Extensibility.

As with FAIR, we define four specific sets of principles to improve best practices in developing mechanistic computational models of biological systems. There is no specific order in the principles, but the acronym CURE seemed appropriate for the topic in question (Fig. 1).

CURE (Another organization devoted to the promotion of curation practices of research compendia also uses the acronym CURE: "Curating for Reproducibility”. However, there is little overlap between the two usages. There is also a UNESCO CuRe curriculum, but this is also unrelated.) covers four key ideas, Credibility, Understandability, Reproducibility, and Extensibility, which we describe in the following sections. These guidelines are meant, where possible, to apply to both machine-readable formats, such as SBML, as well as models distributed in executable code, such as MATLAB, Python or Julia. They also align with previous community efforts to address barriers in comprehensiveness, accessibility, reusability, interoperability, and reproducibility of computational models in systems biology^49^.

### Credible

We use credibility to mean a perceived measure of believability^50,51^. A credible model makes trustworthy and actionable predictions within the range of conditions it was intended to simulate. Prior work on model credibility dates back to 1979^52^, when the Society for Modeling & Simulation International described many concepts associated with model credibility. More recently, several groups within the biological modeling community have discussed model credibility, most notably the ten rules developed by CPMS^53^, devised by the Committee on Credible Practice of Modeling & Simulation in Healthcare (CPMS) in the US, and Musuamba et al.^54^ in Europe, who describe in some detail criteria and concepts that are important to assess the model credibility. Of note, the CPMS working group considered credibility as a descriptor of the practice of modeling and simulation rather than that of a model. Accordingly, the assessment by the ten CPMS rules is geared towards evaluating the practices followed in the modeling efforts^55^. Two core concepts related to model credibility are verification and validation^56^.

Verification is “the process of determining that a model implementation accurately represents the developer’s conceptual description of the model and the solution to the model”^56^. Similar definitions can be found in many other documents^57^. In practice, verification means assessing the correctness of: (i) the model representation (e.g., detecting typographical errors); (ii) the numerical algorithms used to simulate the model (e.g., pseudo-random numbers have the correct distributions); and (iii) the model implementation in software. Models implemented directly in programming languages such as Python may not have (i), and models implemented using standards such as SBML have few concerns about (iii).

When using standards such as SBML, verification involves ensuring that different software applications interpret the description of the model in the same way^58^ and that the software has passed the parts of the SBML test suite relevant for the model^59^. It is worth noting the close correspondence of verification to software testing, including unit and system tests, and documentation^60^. Since models are almost always implemented in software, this should come as no surprise. Static tests can assess whether the model is correct, for example, that the biophysical laws have been entered correctly or that mass balance is not violated. Dynamic tests can offer more subtle checks of the correctness of a model. For example, a model whose variables are concentrations should not be able to reach negative values.

Validation is “the process of determining the degree to which a model is an accurate representation of the real world from the perspective of the intended uses of the model”^56^. Of significant importance is the phrase “intended uses”. All models have a limited scope, that is, they can only make useful predictions within their intended purpose and calibration. This is particularly important for models that might be used in a clinical setting where misuse of a model could have dire consequences. It is important, therefore, that there be a clear statement of the purpose of the model as well as the conditions under which the model is applicable. Validation involves comparing experimental data to predictions made by the model. In practice, given the nature of scientific models, not all model predictions can be validated, and validation is more a measure of confidence in a model’s credibility to match reality than an absolute statement of truth.

Mechanistic models often have parameters whose values are determined through parameter fitting. This is a well-established area, which we will not cover in detail here. Readers are referred to the following articles for further details^39,61–72^.

For parameter-free logic-based models in biology, the credibility lies in the causality of the statements used to build the logical rules and functions^73^, as well as the binarized interpretation and use of small- and larger-scale experimental data^74^.

#### Uncertainty quantification

In recent years, there has been a growing interest in the biomedical community to use uncertainty quantification (UQ) as part of a credibility assessment^75^. UQ has, however, been well established in other scientific domains for many decades^76^ and involves calculating how uncertainty in the experimental data and model parameters contributes to uncertainty in the model outputs. A model that generates highly uncertain outputs is seen as less credible. Verification, validation, and UQ have been collectively referred to by many practitioners using the acronym VVUQ^57^. A recent article by Colebank et al.^71^ reviews elements of this topic.

Another criterion that can enhance a model’s credibility is the provenance of the data used to build the model. Where did the data come from, and was it modified in some way? Table 1 gives a summary of some of the main criteria that can be used to assess credibility. However, not all criteria are equally important, with validation and verification usually being key in this regard. The first four rules in the 10 rules devised by the Committee on Credible Practice of Modeling & Simulation emphasize all these aspects^53^.

**Table 1:** A range of criteria that can establish the credibility of a model

Attribute	Criteria
Validation	How well do the model outputs match reality?
Verification	Has the model been constructed without error? Are the simulation algorithms correctly encoded and operating without error?
Uncertainty	Have the effects of uncertainty in the model outputs been reported?
Provenance	Can the data that was used to calibrate or validate the model be traced to its original source?
Annotation	Have the inputs and outputs of the model been well defined?
Assumptions	Have the assumptions used in the model been made explicit?
Purpose	Has the purpose of the model been adequately described?
Scope	Has the scope, that is, the space within which the model can be used, been specified?
Unbiased	Was the model calibrated on unbiased data? This depends on the scope of the model, but if a model is to be used across a diverse population, then clearly, the calibration data needs to be diverse.

In many cases, modelers may opt not to consider some or all of these criteria in their work, primarily due to the burden of having to do the checks. We, therefore, recommend automating as many of these criteria as possible. For example, verification of SBML-based models can be achieved using the BioSimulation resource^77^. Validation tests could be provided in a standard format as part of the software modeling code, just as software engineers often provide validation tests as part of their distributions^78^. More difficult is including information about data provenance and model assumptions. However, the use of model standards such as SBML or CellML allows models to be annotated with this information. It is more difficult to address the availability of experimental metadata and under what conditions the model was validated. However, work by the synthetic biology community has made progress in this area with the development of protocols to describe an experiment^79^. This is an important area that needs further work, and these guidelines may stimulate further research.

When models have an explicit representation (e.g., SBML) as opposed to just a software implementation, analysis tools can do deep dives into the model to examine its biophysical assumptions. Also, more in-depth verification can be done on the explicit model representation, such as detecting errors in the formulation of biophysical laws^80^. MEMOTE^81^ is a successful example of automated software that can do deep dives into genome-scale models to assess the quality of the model.

Credibility is widely used in software development through the commenting of code, using version control for provenance, and unit and system testing for validation^60^. Verification is achieved through in-depth checking of the software compilers and runtime systems.

### Understandable

One of the aims of the scientific method is to gain an understanding of how the world works by proposing models and theories that can be tested. We understand theories (to paraphrase^82^) to be bodies of knowledge that are broad in scope. Chemical reaction theory or the central dogma in biology are examples of broad scope. In contrast, models are instantiations of theories and, as a result, are narrower in scope and often represent a particular biological process of interest. This includes computational models of metabolism, protein signaling networks, etc. Both models and theories are the most important outcomes of the scientific method. They provide a way to rationalize a set of observations and make predictions about the future state of the system. However, the act of “understanding”^83^ a model or theory is not an easy concept to define and may encompass a number of different aspects. In particular, how might one quantify “understanding”? Philosophically, de Regt and Dieks^83^ define understanding as a given phenomenon if there exists a theory that describes the phenomenon. However, such definitions are hard to quantify. Instead, we will focus on measures that can be used to provide some level of confidence in how understandable a model is.

Biological systems, even small ones, are challenging to understand because of the nonlinear interactions among the components. The problem becomes even more acute as the systems we study grow larger. Biological systems often interact in complicated and nonlinear ways that result in emergent behaviors^84^. For example, no amount of understanding of the components of DNA—purines and pyrimidines, or at a lower scale, carbon and hydrogen atoms—would lead us to predict its complex structure or understand its biological role. The role emerges from the way the components are assembled.

**Fig. 2:**
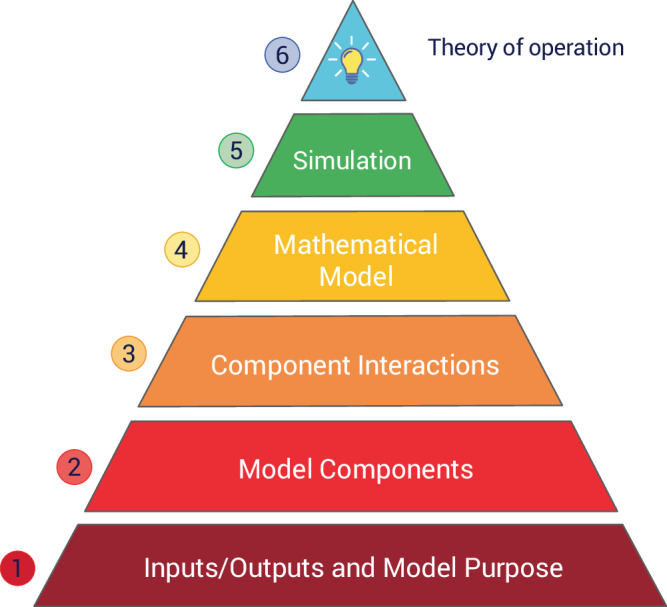
Quantifying understanding through a hierarchy of criteria. The hierarchy begins with the simplest requirements, culminating with a general theory that explains the operation of the model. Not all hierarchical stages may be achieved in any given model.

How can we measure understanding? One way is to divide a model’s components and attributes into levels of perceived importance. For example, we could understand different aspects of a model, such as knowing a model’s purpose, its components, the biophysical laws that describe how the components interact, its inputs, outputs, assumptions, and limitations. Figure 2, depicts a pyramid that organizes such a hierarchy of ‘understanding’. At the base of the pyramid is the most rudimentary understanding; successive layers indicate increased levels of understanding. At the most rudimentary level (1), we want to know the purpose and objectives of the model as well as its inputs and outputs. Subsequent levels include the system components being modeled (2); the interactions between components that are modeled (3); a mathematical description of these interactions (4); a way to evaluate the mathematical model (e.g., solve a system of differential equations) (5); and finally, if possible, a general theory that explains the behavior of the model (6).

Technology is already available to assist in identifying model components (Level 2) and interactions (Level 3), as well as adding metadata to a model (Level 1). Such information can be provided through model annotations^85^. The mathematical model (Level 4) also includes the model’s assumptions, which can be added as annotations using the SBO (Systems Biology Ontology) ontology^86^. For genome-scale metabolic models, the scientific community developed a list of detailed recommendations to annotate such models at Levels 1–4 following an extensive debate at their 2018 annual conference on constraint-based reconstruction and analysis (COBRA)^87^. Scientific models do not have to be mathematical, but for our purposes, most models are, and once a model is defined mathematically, it is often possible to convert the model into an executable form so that simulations can be carried out to generate predictions. Simulation information can be annotated using ontologies such as KiSAO (Kinetic Simulation Algorithm Ontology)^88^. The pinnacle of understanding is Level 6, which is the formulation of a theory that explains the behavior seen in the model.

Level 6 is the most difficult to quantify and involves explaining in terms of both fundamental and higher-order concepts how a given behavior emerges. Making this more difficult is that many biological systems show emergent behavior^84^, where we observe behavior that is not found in any individual component of the system. Even simple systems can reach this threshold. For example, a system of only two components and some non-linearity can show sustained energy dependent oscillations^39,89^. Understanding the individual components is not sufficient to explain the origin of the oscillations^90^ but requires a theory to help understand how, as a system, we observe oscillations. In this particular case, we need additional concepts, such as hysteresis and negative feedback, to understand the origin of the oscillations. For very complex systems, there may be multiple levels of abstraction that describe many levels of emergent behavior. Such abstractions are commonly used in electrical engineering and computer science. This is what makes it possible to engineer these highly complex systems. Reverse engineering abstraction layers in biology is, however, difficult^91,92^ due to the fact that we are dealing with evolved systems that are not always as well structured as our own engineered systems. Unfortunately, no ontology exists to adequately annotate how a system operates. TEDDY (Terminology for the Description of Dynamics) is closest to such an ontology^86^, which can be used to describe dynamics but does not, as yet, have the capacity to describe a theory of operation.

### Reproducible

Reproducibility is a cornerstone of the scientific method, and over the last 10–15 years, much discussion has been devoted to this topic^93,94^; mostly about the lack of reproducibility of many scientific results. Often, we think of wet lab experiments in biology as being difficult to reproducible because they are multifaceted and inherently variable. However, it has also been discovered that computational experiments are often not reproducible^15^. This is surprising given that computation involves well-defined and often deterministic procedures. One thing that has become clear is that community standards such as SBML have significantly improved the reproducibility of computational models by providing a machine-readable representation in a standard format^15,95^. Moreover, recent evidence supports the opinion that reproducible models are more cited and more likely to be reused in subsequent studies^96^. Even if reproducibility is not a high priority for its own sake, verification, validation, and reuse of models require a model to be reproducible^97^. In recent years, we have seen the emergence of software tools and standards that offer support to assist modelers in creating reproducible models^80,98–100^. One could even go as far as to say that in systems biology at least, the problem of reproducibility is solved^16^. We will not revisit the many issues and recommendations concerning the reproducibility of computational experiments, and readers are referred to the many publications that have appeared in recent years^16,17,27,28,51^.

### Extensible and reusable

Science builds on past efforts, standing on the proverbial shoulders of giants. This is no different when building computational models where past computational models can be enhanced, reused, and further validation applied. Unfortunately, many published models cannot be easily reused^53^. This is because many models were published without an explicit representation of the model. Instead, the model is deeply embedded in a software implementation such as MATLAB, Python or Julia^101^. The software implementation adds many complexities (e.g., file interfaces and control logic for computing a solution), often in the form of multiple files with minimal documentation. Moreover, models deployed in this way are in a mathematical representation that loses considerable biological information. For example, when a biochemical pathway model is reduced to a set of differential equations, the stoichiometric structure of the network is either lost or is difficult to reverse engineer. A further concern is that the mathematical representation greatly complicates the ability to query models to discover similar pathways, kinetics, and other characteristics. These considerations are some of the reasons why genome-scale models are published using SBML^19,81^ so as to preserve as much biological information as possible.

Hence, the primary concern with publishing a model solely as its software implementation is that it is difficult to reuse the model, either in part or in whole. For example, combining a model written in MATLAB with a model written in Python can be a costly exercise. Likewise, converting models from one format to another, for example, MATLAB to SBML, can also be error-prone and costly^29^. The use of standards such as SBML offers additional advantages, such as automated deconstruction^36^ and reuse of models^102^ through the use of model annotations or deep analysis using tools such as MEMOTE^81^. Models expressed in SBML are much easier to reuse or extend. When converted to a human-readable language like Antimony^103^, reuse becomes even easier.

Other disciplines employ formats such as Modelica^104^ or representations such as Simulink^105^ to assist in reuse, but such techniques have not been widely used in developing biological models.

If executable code is used to represent models, then the model should ideally be partitioned into reusable program functions with ample documentation to illustrate how to reuse the model and what the various symbols in the mathematical equations represent.

In addition to technical aspects, licensing information is crucial for extensibility and reusability, as it defines if, how, and under what conditions models can be reused—only open and permissive licenses enable unrestricted reuse.

## Recommended requirements

The previous discussion provides a wide range of criteria that can be used to satisfy the CURE guidelines, and fulfilling all the requirements would be quite onerous. For most academic studies, it might be sufficient to meet a small number of criteria. For models that might be used in a clinical setting, it would be prudent to satisfy as many criteria as possible. Organizations such as the FDA (Food and Drug Administration) have already begun to issue guidelines for models used in medical devices^106^. Although these guidelines are not related to systems biology, such efforts point to a growing trend in the biomedical sciences.

**Fig. 3:**
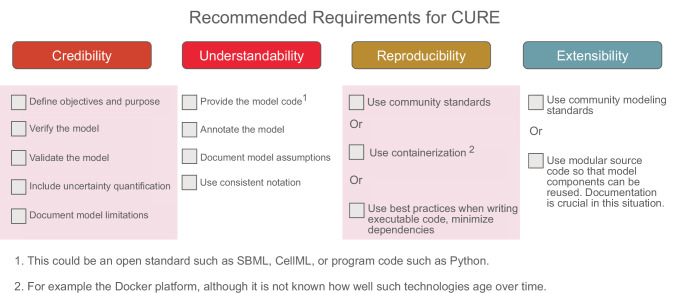
A suggested checklist for CURE that could be used for research-based models. The baseline requirements are highlighted in shaded pink boxes. A score of one out of ten can be given based on the number of criteria met. For example, the baseline requirement will yield a score of 6/10. Note that if publishing models via source code, it is essential to specify the version number of the software platform as well as the version numbers of any dependency packages that were used. The use of containerization platforms such as Docker can sometimes help in these situations.

For academic research, we propose a recommended set of requirements from each aspect of CURE that would be sufficient to significantly impact computational modeling. We provide a checklist in Fig. 3, which also highlights the baseline requirements.

A key requirement is the need to develop standardized approaches to assess and communicate the extent to which a given model, or a modeling study, satisfies the recommended or baseline requirements. An example to consider is the approach taken by the CPMS working group to develop a rubric that considers the extent of outreach to various stakeholders in satisfying various credible practice guidelines^55^. Such evaluations, typically conducted as self-assessments by the respective study authors, can be shared with the community as part of supplementary material in published studies^107,108^. Automation could greatly facilitate this assessment process, especially if provided in advance and used during model development.

### Baseline requirements

If all the recommended requirements are considered too onerous, it is possible to define a baseline requirement. These are the essential or foundational standards that are necessary for basic credibility, though they do not meet the full recommended requirements specified by CURE. The baseline requirement is similar in intent to the recent report^109^ from the US National Academies, which emphasizes purpose, verification, validation, UQ, and reproducibility, though their statement on reproducibility is vague. We include scope and model limitations into our baseline, which the National Academies documents do not explicitly mention, though it could be considered part of the statement of purpose. The baseline requirements are highlighted in Fig. 3, and a more detailed summary is given in Table 2.

**Table 2:** Summary of recommended and baseline requirements

**Credibility (baseline requirement):**	
1.	Clearly define the objectives and scope of the modeling study, including the biological question being addressed and the specific hypotheses to be tested.
2.	Where possible, verify the model by checking it with other simulators. Use model-checking tools to identify errors in the model^60^.
3.	Validate the model against experimental data using accepted statistical procedures to assess model accuracy and predictive power.
4.	Where possible, assess how sensitive the model is to uncertainty (UQ) in parameter estimates, model structure, inputs, and assumptions.
5.	Clearly document the limitations of the model, including areas where assumptions may not hold or where uncertainties exist, to provide context for interpreting results and guiding future research.
**Understandability:**	
1.	Provide a representation of the model that is both machine-readable and human-readable. Ideally, this should be in an open community standard and be an explicit representation of the model that is not intertwined with control logic, file input/output, and other implementation details.
2.	Provide comprehensive documentation that explains the model structure, equations, and parameter values, e.g., by following the suggestions by Carey et al.^87^. When using open community standards such as SBML or CellML, submit the models to a recognized model repository. If the model is expressed in a programming language, deposit your model code at repositories such as GitHub, BioModels^24^, or ModelDB^42^.
3.	Where possible, annotate the model to clarify any ambiguous terminology. When using a programming language to express a model, use commenting to annotate the model.
4.	Use consistent notation and terminology to ensure consistency and clarity in model descriptions. Where possible, follow common notations used in the community.
5.	Try to document all assumptions made during model development, including simplifications, approximations, and parameter values, to provide transparency and facilitate reproducibility.
6.	Try to provide a clear graphical illustration of the model. If the model is a biochemical network, then use machine-readable formats such as SBGN^113^, preferably using a community modeling standard such as SBML Layout^114^ and Render^115^.
**Reproducibility (baseline requirement):**	
1.	Follow established standards and guidelines for model development, such as the SBML and Minimum Information Requested in the Annotation of Models (MIRIAM), to enhance interoperability and facilitate model sharing and exchange. If using executable code, make sure best practices for code development are used.
2.	Embrace and promote open science practices by openly sharing publicly funded models, data, and code with the scientific community, promoting transparency, reproducibility, and collaboration.
**Extensibility and reuse:**	
1.	Use open modeling standards where possible. If using executable code such as Python, separate the model code from the runtime code so that, in principle, the model can be reused by other Python users. Care should be taken when describing the components of the model as comments. One approach is to provide the model as a software function that can be called by other code. Try to use open-source licensing so that there are no restrictions on the reuse of the research.
2.	If a model is represented using a modeling format such as SBML, reuse should be much easier since the model is expressed in biological terms. If the model is annotated, then automated systems can be devised to automate the merging and disassembly of models into individual parts or portions for reuse.
3.	Provide licensing information and use open and permissive licenses.

## Conclusion

This paper introduces a set of principles for developing robust, credible biological models. These guidelines are meant to complement the existing but more generically applicable FAIR guidelines. As with FAIR, we propose a four-word moniker, CURE, that defines four core attributes of good modeling practice. These include credibility, understandability, reproducibility, and extensibility.

The need for CURE models is highlighted by recent interest in Biomedical Digital Twins, a technology that will rely on having robust models of biomedical systems^5,109,110^, but it is clear that such guidelines would also benefit the wider biological modeling communities^111^.

For credibility, we recommend the use of verification, validation, and UQ; for understandability, we discuss the clarity of model descriptions and the importance of annotations; for reproducibility, our focus is standards and open science practices; and for extensibility, we emphasize open standards and the use of modular models. We outline recommended requirements for each guideline and propose a baseline level below the recommended requirements that is largely in alignment with the National Academies report^109^.

While all of the CURE principles are important, we wish to highlight credibility, the first principle. Credibility is the degree to which a model can be trusted when applied to a given problem. In a biomedical application where concern is with patients and their well-being, the trustworthiness of a predictive model is paramount. Although tangential to models used in systems biology, the FDA recently published^106^ a guidance document on assessing the credibility of computational models, but applied only to medical devices. Many of their recommendations relate to model verification and validation, but also contain aspects related to model plausibility, which considers the plausibility of the governing equations, assumptions, and model parameters. The FDA document also emphasizes UQ for estimating uncertainty in the model outputs. These are considered foundational for credible modeling. However, these have not yet significantly infiltrated the biomedical modeling community. The recent National Academies^109^ report on Digital Twins emphasizes the same points. The report also stresses the critical need for data and modeling standards to enable interoperability and reuse. As an initial effort to support VVUQ, the BioSimulations resource^77^ provides a verification service where a given model can be run against multiple independent simulators to help verify the simulation engines.

Finally, automation is key to making the CURE guidelines practical to reduce the burden on practitioners and accelerate wider adoption. To be clear, by automation, we don’t mean automating model construction, although one could envisage such a scheme. Instead, we are referring to automating model checking as outlined by CURE once the model has been built.

By automation, we do not mean automating model construction, although one could envisage such Automation of these guidelines on models that are published in arbitrary programming languages will be almost impossible unless modern AI can in some way be leveraged. More realistic is automation when models are distributed using open standards that have a strict formal structure and are designed to be computer-readable. One project that has been successful in automating model checks is MEMOTE^81^. This software is able to do deep dives into genome-scale metabolic models (GEMs) to assess the quality of the model. This is possible because GEMs are stored in computer-readable form (SBML). Other efforts underway include verification of rate laws (https://github.com/sys-bio/ratesb_python) and, as yet, an unpublished service at (https://github.com/biosimulators/bsvs) that can be used to verify an SBML model by running a given model against multiple simulators. For model calibration, PEtab^112^ can be used to describe the model calibration workflow, including UQ. This allows model calibration to be reproducible and therefore automated. The PEtab community is currently developing version 2.0, which will expand its capabilities even further. PEtab will be an important component of an automated system. However, other open standards remain to be developed. In particular, automated model validation will be a key requirement to automate CURE guidelines.

## Data Availability

No datasets were generated or analyzed during the current study.
